# Input-to-output transformation in a model of the rat hippocampal CA1 network

**DOI:** 10.3389/fncom.2012.00057

**Published:** 2012-08-06

**Authors:** Andrey V. Olypher, William W. Lytton, Astrid A. Prinz

**Affiliations:** ^1^School of Science and Technology, Georgia Gwinnett CollegeLawrenceville, GA, USA; ^2^Department of Physiology and Pharmacology, SUNY DownstateBrooklyn, NY, USA; ^3^SUNY Downstate/NYU-Poly Joint Biomedical Engineering ProgramBrooklyn, NY, USA; ^4^Neural and Behavioral Science Program, SUNY DownstateBrooklyn, NY, USA; ^5^Department of Neurology, SUNY DownstateBrooklyn, NY, USA; ^6^Kings County HospitalBrooklyn, NY, USA; ^7^Department of Biology, Emory UniversityAtlanta, GA, USA

**Keywords:** hippocampus, detailed neuronal models, information processing

## Abstract

Here we use computational modeling to gain new insights into the transformation of inputs in hippocampal field CA1. We considered input-output transformation in CA1 principal cells of the rat hippocampus, with activity synchronized by population gamma oscillations. Prior experiments have shown that such synchronization is especially strong for cells within one millimeter of each other. We therefore simulated a one-millimeter *patch* of CA1 with 23,500 principal cells. We used morphologically and biophysically detailed neuronal models, each with more than 1000 compartments and thousands of synaptic inputs. Inputs came from binary patterns of spiking neurons from field CA3 and entorhinal cortex (EC). On average, each presynaptic pattern initiated action potentials in the same number of CA1 principal cells in the patch. We considered pairs of similar and pairs of distinct patterns. In all the cases CA1 strongly separated input patterns. However, CA1 cells were considerably more sensitive to small alterations in EC patterns compared to CA3 patterns. Our results can be used for comparison of input-to-output transformations in normal and pathological hippocampal networks.

## 1. Introduction

Normal hippocampal functioning is essential for learning and memory. Abnormalities of the hippocampus are associated with the cognitive symptoms of schizophrenia, Alzheimer disease and other disorders (Disterhoft et al., 2004; Walker et al., 2006; Schobel et al., 2009). Hippocampal information processing and its contribution to cognitive symptoms has been described using several animal and computer models: learning and reproducing sequences of stimuli (Hasselmo et al., 2002; Gluck et al., 2003; Cutsuridis et al., 2010), properties of place fields in rodents (Olypher et al., 2006), generation of population rhythms, such as gamma oscillations, associated with cognitive processes (Neymotin et al., 2011; Volman et al., 2011), and information transmission in the hippocampus, in particular from the hippocampal field CA3 and entorhinal cortex (EC) to CA1 (Treves and Rolls, 1994; Schultz and Rolls, 1999).

In this work, we characterized information processing in the hippocampus somewhat differently. We have assessed how differences between input patterns to hippocampal field CA1 are transformed into differences between the corresponding output patterns of CA1 spiking activity. In contrast to classical estimates of information transmission as for example in Treves and Rolls (1994), our approach does not involve characterization of ensemble probabilities of spiking distributions in presynaptic and postsynaptic networks.

The main goal of the present study was to quantify input-to-output transformation performed by a set of gamma-synchronized CA1 principal cells. This problem is important since hippocampal field CA1 is a main output region of the hippocampus (Amaral and Lavenex, 2006), and gamma oscillations (30–100 Hz) are characteristic for exploratory behaviors in rodents (Bragin et al., 1995; Csicsvari et al., 2003) and cognitive processes in monkeys (Jutras and Buffalo, 2010).

Using morphologically and biophysically detailed neuronal models is essential for the analysis of this problem. Simplified models do not discriminate possible differences between patterns of synaptic activation. There are a number of tested realistic models of individual CA1 neurons; see e.g., (Graham, 2001; Li and Ascoli, 2006; Katz et al., 2009). We used one such model from (Jarsky et al., 2005). The model had 1330 compartments and thousands of excitatory and inhibitory synapses. We simulated information processing in a set of 23,500 such models. The size of the set is an estimate of the number of CA1 principal cells that spike synchronously during population gamma oscillations in the rat CA1 (Lubenov and Siapas, 2009; Sabolek et al., 2009).

We studied how CA1 principal cells discriminate patterns of spiking activity of presynaptic principal cells in the hippocampal field CA3 and EC. CA1 receives inputs from CA3 by the Schaffer collaterals and from EC by the perforant path (Amaral and Lavenex, 2006). The average level of spiking activity in the CA1 principal cells caused by input was the same for all inputs considered. Intuitively, if an input pattern makes a neuron spike then the neuron should also spike in response to similar patterns—otherwise, neurons would be too sensitive to noise. On the other hand, neurons should discriminate between sufficiently different input patterns to spike selectively. To determine discriminative properties of the network we quantified its binary output in response to four categories of inputs: similar and distinct activity patterns of hippocampal field CA3 presynaptic cells, and similar and distinct activity patterns of presynaptic cells from the entorhinal cortex.

We found, in particular, that the CA1 network output was more sensitive to small differences between patterns of presynaptic activity in EC than to small differences between patterns of presynaptic activity in the hippocampal field CA3. CA3 and EC input patterns that had average difference were processed by the CA1 network similarly. Our results can be used in comparative studies of normal and abnormal information processing in CA3, EC, and CA1 to reveal how neuronal and network abnormalities translate into cognitive disorders.

## 2. Methods

### 2.1. Neuronal model

We used a model based on a reconstructed morphology of a CA1 principal cell and known biophysical properties of such cells. The model has been described in Jarsky et al. (2005) and is publicly available on ModelDB (https://senselab.med.yale.edu/modeldb/). The model neuron has 23,424 excitatory and 2465 inhibitory synapses that are distributed over the compartments according to location-specific densities described in Megias et al. (2001).

**Figure 1:**
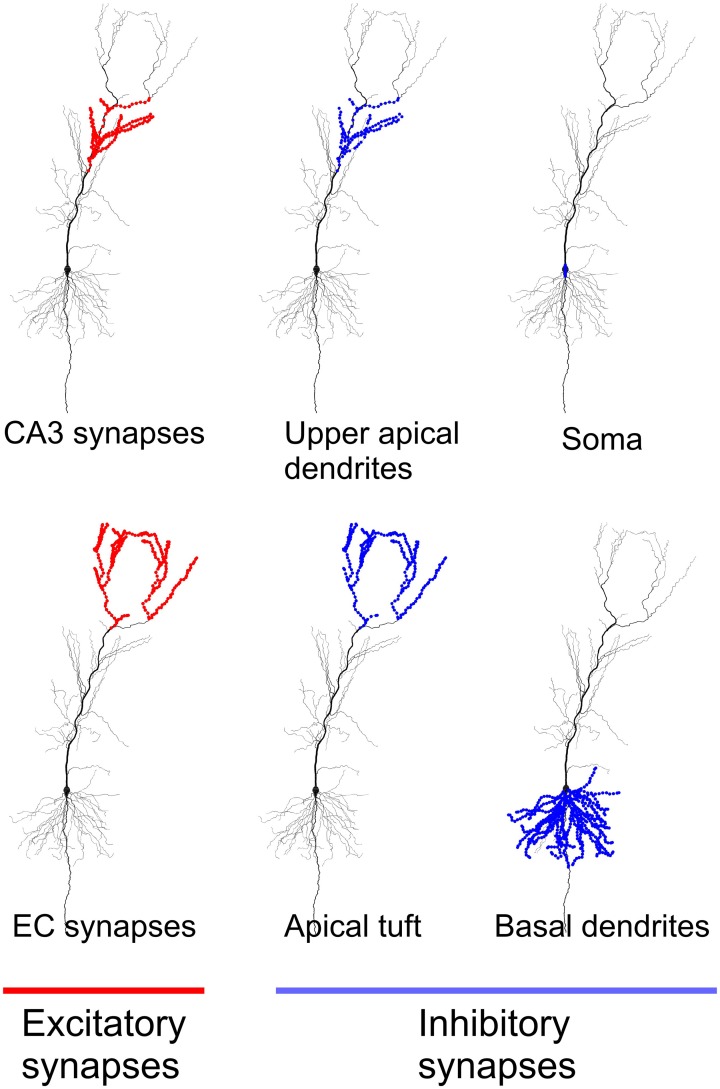
**Location of excitatory and inhibitory synapses in the neuronal model used in the study.** All excitatory synapses located in apical dendrites were considered synapses from CA3 principal cells. All excitatory synapses located in apical tuft were considered synapses from EC principal cells.

Figure 1 shows locations of the excitatory and inhibitory synapses of the model used in the study. As in Jarsky et al. (2005) 4407 excitatory synapses located in the upper apical dendrites were considered CA3 synapses, and 1918 excitatory synapses located in the most distal branches of apical dendrites and dendritic tuft were considered EC synapses. The selection of inhibitory synapses is described in the next section on network inputs.

A realistic morphology was essential for our study. Similar synaptic activation patterns with the same local densities and total numbers of activated synapses may or may not cause action potential initiation in the model neuron depending on fine details of input localization (Gasparini et al., 2004). Models with simpler morphology cannot capture fine distinctions between input patterns.

Besides passive membrane properties, the model has a *Na*^+^ conductance, and delayed rectifier and A-type *K*^+^ conductances. As in Jarsky et al. (2005), we considered only AMPA and GABAa synapses. The model was provided with strong dendritic excitability implemented by a linear increase of the sodium conductance with distance from the soma; see details in Jarsky et al. (2005). We used *exactly* the same parameters of the model as in Jarsky et al. (2005). The model was simulated using the NEURON environment (Hines and Carnevale, 1997).

### 2.2. Network inputs

We modeled information processing in a square millimeter patch of CA1 during population gamma oscillations. In the model, we used 23,500 CA1 principal cells. This is an estimate of the number of principal cells in a square millimeter patch given that there are approximately 320,000 principal cells in 13.6 square millimeters of the whole rat CA1 (Bernard and Wheal, 1994; Bernard et al., 1997). We assumed that at the beginning of a 20 ms gamma period, all CA1 principal cells were in the same state. We hypothesized that this reset was brought about by CA1 inhibitory interneurons involved in population gamma (Csicsvari et al., 2003). We did not model connectivity between the CA1 principal cells (Orman et al., 2008) since we were only assessing immediate (wave-front) responses that did not depend on principal cell interaction. The cells in the model represented a network processing information in the sense that they shared some inputs.

**Figure 2:**
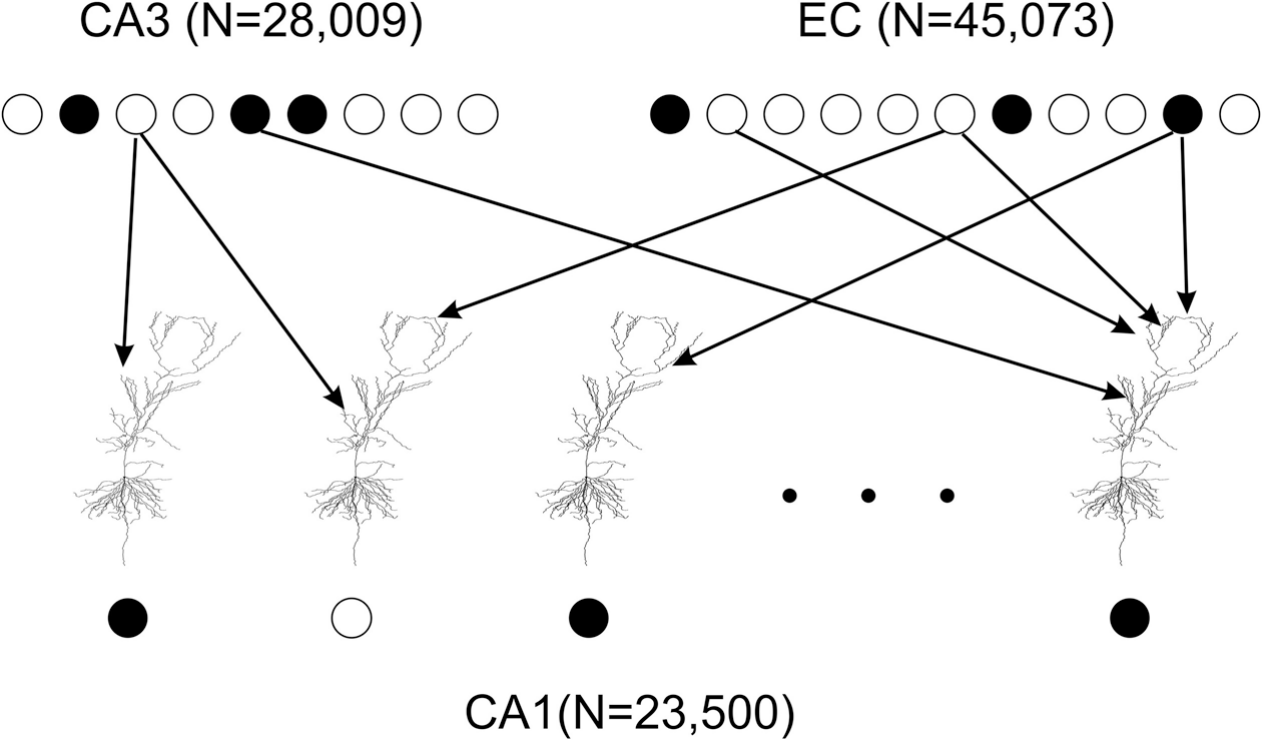
**Connectivity in the model of the CA1 network.** 23,500 principal cell models represent a one square millimeter patch of CA1. All neuronal models are identical. However, each model has unique random mapping between its 1918 dendritic tuft synapses and EC cells and between its 4407 upper apical and oblique dendrite synapses and CA3 cells. Spiking EC and CA3 cells (filled circles) may or may not initiate an action potential (filled circle) in a model CA1 principal cell within a 20 ms time window. Thus, the CA1 network model transforms a binary vector of a EC and CA3 input into a binary vector of CA1 activity output.

To model excitatory inputs to CA1 principal cells we adopted the following assumptions. First, each presynaptic cell in EC and CA3 had at most one synaptic contact with an individual CA1 principal cell (Li et al., 1994). Second, presynaptic cells for each CA1 cell were chosen randomly according to a uniform distribution; the connectivity did not change over the course of simulations (Figure 2). With regards to EC presynaptic neurons we assumed that each of them targeted 1000 CA1 cells in the patch (Solstad et al., 2006). Since the CA1 neuronal model has 1918 EC synapses, we got 23,500 × 1918/1,000 = 45,073 presynaptic EC cells. The estimated average number of postsynaptic CA1 targets for an individual CA3 principal cells is 18,000 (Ascoli and Atkeson, 2005). Data and modeling suggest that locations of CA1 cells targeted by a CA3 cell are distributed non-uniformly (Bernard and Wheal, 1994; Ropireddy and Ascoli, 2011). We set ~20% of CA3 projections to target the patch. Given 4407 CA3 synapses in the model, this yielded in 28,009 presynaptic CA3 principal cells. Activity patterns of EC and CA3 presynaptic cells were therefore represented by binary vectors of dimensions 45,073 and 28,009 respectively. Each vector component was equal to one if the corresponding presynaptic cell spiked, and to zero otherwise. Presynaptic activation was simultaneous.

We divided inhibitory synapses onto CA1 principal cells into two categories. The first category of inputs provided feedback inhibition, which reset all principal cells in the modeled patch. We modeled these inhibitory inputs implicitly by starting all CA1 neuron models in the same initial state to mimic the gamma reset. The second category of inputs provided feedforward inhibition. Corresponding inhibitory inputs mostly come from bistratified and oriens lacunosum—moleculare (OLM) interneurons (Klausberger et al., 2003; Klausberger and Somogyi, 2008). Bistratified interneurons target basal and oblique dendrites of principal cells, OLM interneurons target the dendritic tuft (Figure 1). Bistratified and OLM interneurons fire several milliseconds earlier than principal cells (Klausberger and Somogyi, 2008). On this basis we assumed that inhibitory synapses from these interneurons were activated approximately simultaneously with excitatory synapses to principal cells. Synaptic conductances were modeled with double-exponential functions: τ_1_ = 0.2 ms, τ_2_ = 2.0 ms for AMPA synapses, and τ_1_ = 1.0 ms, τ_2_ = 18.0 ms for GABAa synapses. Synaptic reversal potentials were 0.0 mV and −75.0 mV, respectively. The model had in total 120 inhibitory synapses in upper apical and oblique dendrites, 333 in the dendritic tuft, 1447 in the basal dendrites, and 44 in the soma. We used the following levels of activation of inhibitory synapses: upper apical and oblique dendrites, basal dendrites, and soma −50%, dendritic tuft −20% out of the corresponding total numbers. The levels of activation were chosen to match the results of simulations in Jarsky et al. (2005) (see their Figure 2).

### 2.3. Metric for the network inputs and outputs

To quantify differences between pairs of binary patterns of presynaptic activity (CA1 network inputs) we used the Hamming distance. Each input pattern produced a spiking pattern in CA1 (CA1 network output). We quantified differences between the output patterns also using the Hamming distance. Hamming distance, HD(x, y), between two binary vectors *x* and *y* is equal to the number of vector components that are not the same. For example, if *v*_0_ = (0, 0, 0, 0, 0), *v*_1_ = (0, 1, 0, 1, 1), and *v*_2_ = (0, 1, 1, 1, 0) then HD(v_0_, v_1_) = 3 and HD(v_1_, v_2_) = 2. If a CA1 cell spiked within 20 ms after synaptic activation the corresponding component of the output vector was set to one, otherwise the component was set to zero.

### 2.4. Simulations

Simulations were performed on a 256 dual-socket/dual-core AMD 2.6GHz-based Sun X2200 compute nodes cluster at Emory University and 1120 Intel Xeon Quad-Core 2.33GHz-based compute nodes of the Union Square (USQ) cluster at NYU. No parallelization of computations was used. For any input, the neuronal model was simulated for no longer than 20 ms (a typical period of gamma oscillations) of the model's time. If the somatic membrane potential reached 10 mV (action potential threshold) within 20 ms the simulation stopped and the input was considered as initiating an action potential. Otherwise, the input was considered as not initiating an action potential. For simulations, we used NEURON's multiple order variable time step integration method *CVODE*. Since we were not interested in accurate spike waveforms it was sufficient to set the local absolute error tolerance equal to 10^−4^. On average, a simulation of one input pattern to one neuronal model took approximately 7 s of processor time. Analyses of simulation data were done with Matlab (Mathworks, Inc.).

## 3. Results

### 3.1. Activity levels in EC, CA3, and CA1

**Figure 3:**
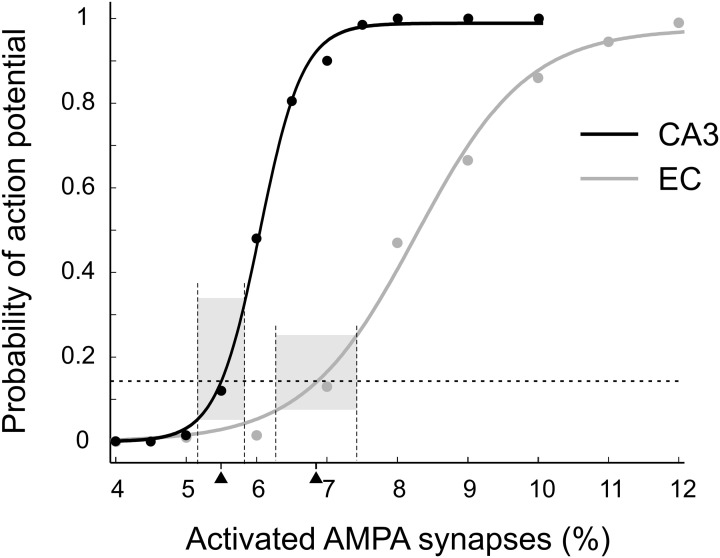
**Probability of action potential initiation in the CA1 principal cell modeled as a function of excitatory drive.** EC inputs (gray), CA3 inputs (black). Each point is the portion of 200 random patterns of activated synapses that initiated an action potential. Dashed horizontal line indicates the target action potential probability of 0.14. This probability was achieved when 5.5% of CA3 or 6.85% of EC synapses were activated (black triangles). The bases of the gray boxes centered on the triangles show theoretical variations of activated synapses in the input patterns (see details in the text). The heights of the boxes show corresponding variations in the probabilities of action potential initiation.

We adjusted presynaptic activity to conform to the results of Brun et al. (2002, 2008). These studies showed that CA1 place cells have a peak firing rate of about 7 Hz with only CA3 inputs or only EC inputs, corresponding to a ~14% probability of action potential initiation (API) during a 20 ms gamma period. To find the level of presynaptic activity resulting in 14% API probability in CA1 principal cells we calculated CA1 outputs for multiple levels of CA3 and EC activity (Figure 3). For each activity level we simulated 200 patterns. Fits with Boltzmann functions showed that 14% activity in CA1 (3290 spiking neurons) was achieved when 1540 (5.50%) out of *N*_CA3_ = 28,009 or 3086 (6.85%) out of *N*_EC_ = 45,073 cells spiked in an input pattern. We simulated only CA3 and EC activity patterns with those numbers of spiking cells.

Under the assumption that the binary patterns that represent activity in inputs and CA1 networks within short time windows are formed by independently spiking neurons, the Hamming distances (HDs) between such patterns have binomial distributions. Consider a pair of CA1 activity patterns, *P*^1^_CA1_ and *P*^2^_CA1_. The Hamming distance between the patterns is equal to the sum of the Hamming distances between components, *P*^1^_CA1,*k*_ and *P*^2^_CA1,*k*_, *k* = 1,…, *N*_CA1_, of the patterns: $\text{HD}(P_{\text{CA}1}^{1},P_{\text{CA}1}^{2})=\sum_{k=1}^{N_{\text{CA}1}}\text{HD}(P_{\text{CA}1,k}^{1},P_{\text{CA}1,k}^{2})$. From the assumption of independent neuronal spiking it follows that the Hamming distance between any pair of components is an independent random variable that is equal to one or zero. Since there are two combinations, (0, 1) and (1, 0) with the Hamming distance equal to one, the probability that the Hamming distance between two components is equal to one, *pr*HD_CA1_ ≡ Prob{HD(*P*^1^_CA1,*k*_, *P*^2^_CA1,*k*_) = 1} depends on the probability of CA1 action potential initiation, *pr*API_CA1_ = 0.14, according to the formula *pr*HD_CA1_ = 2 × *pr*API_CA1_ × (1 − *pr*API_CA1_) ≈ 0.24. Hence, HD(*P*^1^_CA1_, *P*^2^_CA1_) follows a binomial distribution with parameters *N*_CA1_ = 23,500 and *pr*HD_CA1_ = 0.24. Accordingly, the mean value of HD(*P*^1^_CA1_, *P*^2^_CA1_) is equal to *N*_CA1_ × *pr*HD_CA1_ = 5640, and the standard deviation is equal to $\sqrt{N_{\text{CA1}}\times pr{\text{HD}}_{\text{CA1}}\times (1-pr{\text{HD}}_{\text{CA1}})}=65$. The mean value implies that on average, only *N*_CA1_ × *pr*API_CA1_ − *mean* [HD(*P*^1^_CA1_, *P*^2^_CA1_)]/2 = 3290 − 5640/2 = 470 out of 3290 spiking neurons spike in both of two randomly selected CA1 patterns. Note also that the mean Hamming distance 5640 is much closer to its maximal value 2 × 3290 = 6580, which corresponds to the case when there is no overlap between two patterns, than to the minimal Hamming distance, which is equal to zero and corresponds to the case when the two patterns are identical.

Similar analysis holds for spiking activity patterns in CA3 and EC. Hamming distances between pairs of random CA3 activity patterns with the probability of CA3 spiking equal to 0.055 are binomially distributed with the parameters *N*_CA3_ = 28,009 and *pr*HD_CA3_ = 2 × 0.055 × (1 − 0.055) = 0.10. The distribution has the mean 2910 and standard deviation 51. In the case of EC input patterns, the binomial distribution has the parameters *N*_EC_ = 45,073 and *pr*HD_EC_ = 2 × 0.0685 × (1 − 0.0685) = 0.13. It has the mean 5750 and standard deviation 71.

### 3.2. Distinct patterns of CA3 and EC spiking

**Figure 4:**
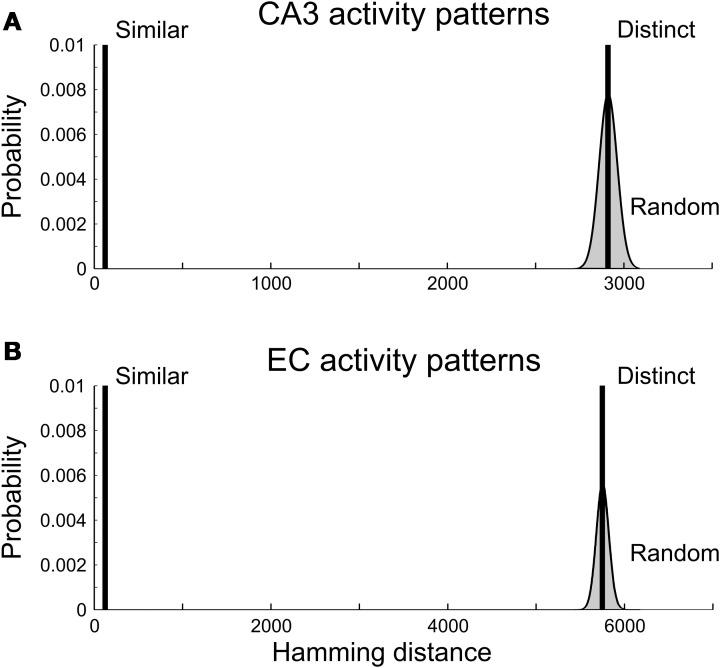
**Hamming distances between presynaptic activity patterns. (A)** CA3 activity patterns. **(B)** EC activity patterns. Vertical bars are truncated; they reach near 1 and represent sets of pattern pairs with equal Hamming distance. Smooth curves bordering gray areas are the normal approximations for the binomial distributions of Hamming distances between all pairs of CA3 **(A)** and EC **(B)** activity patterns with 1540 (CA3) and 3086 (EC) spiking neurons. Pairs of “similar” activity patterns had Hamming distances equal to 2% of the maximal possible values of the Hamming distance for patterns. Pairs of “distinct” activity patterns had Hamming distances equal to the mean values of the binomial distributions.

A pair of spiking patterns was referred to as “distinct” if the patterns had little overlap (high Hamming Distance). For each input group, we generated 20 pairs of distinct patterns; pairwise HD = 2910 for CA3 and HD = 5750 for EC (Figure 4). On average, the generated patterns initiated action potentials in 3316 ± 52 (mean ± standard deviation) CA1 neurons for CA3 inputs and 3313 ± 56 CA1 neurons for EC inputs. In both cases, the mean value of the CA1 spiking probability was close to the target value of 0.14.

We expected that our CA1 network models would transform CA3 and EC inputs differently. One factor was the known difference in spatial summation in CA1 principal cells for activated CA3 synapses compared to EC synapses (Kali and Freund, 2005; Li and Ascoli, 2006). Another factor was the difference between the variation of API probability for the two categories of input patterns. All inputs initiated action potentials in CA1 with the same mean probability of 0.14. However, the number of activated CA3 synapses followed the binomial distribution with the relative standard deviation of $\sigma_{\text{CA3}}/N_{\text{CA3}}\times 100\%=\sqrt{0.055\cdot (1-0.055)/4407}\times 100\%=0.34\%$ in contrast to 0.58% for the number of activated EC synapses. The difference between deviations resulted in a notable difference in the API probabilities (Figure 3, gray bars).

**Figure 5:**
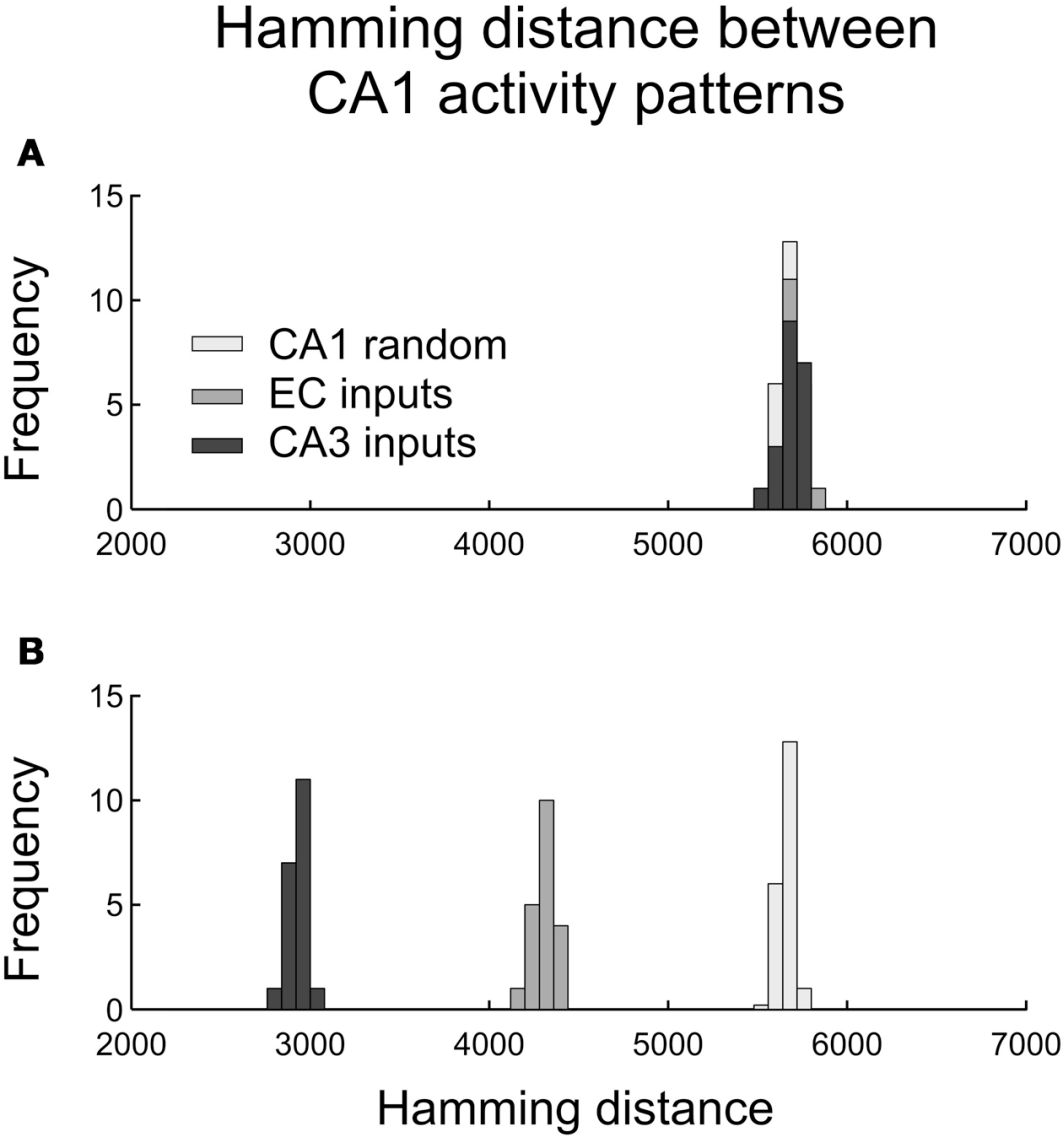
**Histograms of Hamming distances between pairs of CA1 output activity patterns caused by pairs of CA3 and EC presynaptic activity patterns. (A)** Pairwise output Hamming distances in response to pairs of distinct presynaptic patterns (as defined in the text). **(B)** Pairwise output Hamming distances in response to pairs of similar presynaptic patterns (as defined in the text).

Contrary to expectation, we observed no difference in the distributions of the Hamming distances between pairs of CA1 spiking activity vectors for *distinct* CA3 and EC inputs (Figure 5A). The CA1 outputs had mean (HD) = 5688, std(HD) = 56 for CA3 inputs and mean (HD) = 5696, std (HD) = 63 for EC inputs. Those distributions were close to the distribution of the Hamming distances between 20 pairs of random CA1 spiking patterns with 0.14 × 23,500 = 3290 spikes in each pattern (the histograms in light gray in Figures 5A,B). That distribution had the average 5640 and standard deviation 65.

### 3.3. Similar EC and CA3 inputs

The network did transform EC and CA3 input patterns differently when the inputs in pairs were relatively similar to each other (Figure 5B). We generated 20 pairs of CA3 input patterns with the Hamming distance for each pair equal to 2% of its maximal value 3080. In each pair, the two patterns were different in only 62 components. Those patterns, on average, initiated action potentials in 3296 CA1 neurons with standard deviation equal to 43. Similarly, we generated 20 pairs of “similar” EC input patterns with the Hamming distance for each pair equal to 2% of its maximal value 6172. Those patterns, on average, initiated action potentials in 3307 CA1 neurons with standard deviation equal to 52. The two categories of inputs led to pairwise CA1 output patterns significantly closer to each other compared to randomly selected pairs of the CA1 output patterns with 14% of spiking neurons. Namely, for CA3 inputs, CA1 output pattern pairs had mean (HD) = 2931, std (HD) = 50; for EC inputs, CA1 output pairs had mean (HD) = 4315, std (HD) = 63. Similar CA3 inputs led to much closer CA1 outputs compared to similar EC inputs. In other words, the CA1 network model was more sensitive to small differences in EC inputs than to small differences in CA3 inputs.

## 4. Discussion

We studied information flow in a subnetwork of CA1 principal cells that were synchronized by gamma population rhythm. Random input patterns had the same number of spiking presynaptic cells, but differed in the pattern of which presynaptic cells spiked. The patterns produced action potentials in CA1 cells with the same average probability. We found that the CA1 cells were considerably more sensitive to variations in EC spiking patterns than to variations in CA3 spiking patterns. Our results showed that the CA1 network “orthogonalized” not only CA3 memories (Treves and Rolls, 1994) but also, and to a greater extent, EC inputs. This orthogonalization did not require CA1 to outnumber presynaptic networks as in Treves and Rolls (1994).

### 4.1. Neuronal and network models

We used the model of a rat CA1 principal cell developed in Jarsky et al. (2005). This model incorporated experimentally-based biophysical properties and synaptic densities. The 1330 compartments of the model closely approximated the original anatomically-reconstructed morphology. Although such detailed models have been studied for over decade [e.g., (Graham, 2001; Li and Ascoli, 2006; Katz et al., 2009)], they have not been used for simulating networks (Brette et al., 2007). In network simulations of a comparable scale (tens of thousands of neurons), neurons had orders of magnitude fewer synapses and/or compartments; see e.g., (Morgan and Soltesz, 2008).

Computer simulations of hippocampal networks usually fall into two categories. One category is concerned with population dynamics such as oscillations, waves, etc. (Neymotin et al., 2011; Volman et al., 2011). The other focuses on mechanisms of learning and memory (Hasselmo et al., 2002; Gluck et al., 2003; Cutsuridis et al., 2010). We modeled aspects of both of these here. We did not model plasticity but instead assumed that the model neurons already had their memories encoded by synaptic weights. Similarly, we did not explicitly model oscillations, but postulated that the the neurons were synchronized via inhibitory modulation associated with gamma oscillations. Our model demonstrated how CA1 principal cells could transform their inputs within one gamma cycle.

This focus on the single gamma cycle determined the network size and organization of its inputs in our model. Gamma oscillations are highly synchronous between nearby (≤1 mm) CA1 locations (Lubenov and Siapas, 2009; Sabolek et al., 2009). We therefore assumed that all principal cells in one square millimeter patch of CA1 received their synaptic inputs within a short time window, common to all the cells in the patch, and spiked or did not spike in response to those inputs within the same window. We also assumed that inhibition reset all the cells in the patch so that at the beginning of each time window they were all in the same state. Because of those assumptions we did not explicitly model complex inhibitory processes in CA1, lateral interactions between CA1 principal cells, and spike-timing dependent plasticity.

Alternatively, the results of our simulations can also be interpreted as showing how an average CA1 principal cell discriminates differences between input patterns. If considering a single CA1 cell instead of a network, we would have to generate a representative sample of similar and distinct patterns of synaptic activation for this cell. The choice of those patterns would not be trivial given the combinatorics. The target 14% probability of action potential initiation in the model is achieved when either 1540 (5.5%) of 28,009 CA3 or 3086 (6.85%) of 45,073 EC presynaptic neurons spike. The corresponding numbers of combinations of activated synapses are of the orders 10^405^ and 10^206^, respectively. Out of those combinations, we would need to select the patterns that have overlaps consistent with the connectivity between CA3, EC, and CA1. The network approach that we used provided a natural way of selecting appropriate patterns of synaptic activation, and also corresponds to population signaling in the brain.

The results of our simulations are expected to be robust to moderate changes in morphological, biophysical, or synaptic properties. In a comparable study, Jarsky et al. (2005) simulated three neuronal models, utilizing identical biophysical properties with different morphologies. They found that the main input-output characteristics for the three models were similar (Jarsky et al., 2005). CA1 cells with substantially different properties might produce quite different input-to-output transformations. Such different transformations might, for example, occur in the context of distinct populations of CA1 principal cells (Senior et al., 2008; Deguchi et al., 2011; Mizuseki et al., 2011).

### 4.2. Pattern discrimination

In this study, we considered presynaptic activation patterns that initiated action potentials at a selected probability. This is different from prior studies that were focused on how changes in synaptic activation patterns changed spike probabilities in CA1 [e.g., (Li and Ascoli, 2006)]. Given that CA1 principal cells can process CA3 and EC inputs separately (Brun et al., 2002, 2008; Mizuseki et al., 2009), we simulated responses of CA1 principal cells to four categories of input patterns: similar CA3 and EC activity patterns, and distinct CA3 and EC activity patterns.

Our main result is the finding of a new qualitative distinction between transformation of CA3 compared with EC inputs in CA1. Our CA1 network model separated distinct CA3 inputs and distinct EC inputs similarly. In contrast, it enhanced contrast between similar EC inputs far more than it enhanced contrast between similar CA3 inputs. In other words, the CA1 network model was more sensitive to distinctions in EC activity patterns than to distinctions in CA3 activity patterns.

A two percent difference (relative to maximal Hamming distance) across two CA3 patterns leads to an almost 50% difference in the two corresponding CA1 output patterns. This is consistent with the idea of Treves and Rolls (Treves and Rolls, 1994) that CA1 orthogonalizes CA3 inputs to prevent confusion between similar memories. Their proposition was deduced under the assumptions that (1) CA1 preserves the information in CA3 spiking patterns and (2) there are considerably more CA1 principal cells than CA3 principal cell. Our simulations showed that orthogonalization could be achieved with the number of CA1 principal cells (23,500) less than the number of CA3 principal cells (28,009). The simulations also showed that EC spike patterns were notably more separated than CA3 patterns, suggesting that the CA1 system transforms inputs to outputs differently during retrieval compared to learning (Hasselmo et al., 2002).

A possible explanation for the difference between CA3 and EC inputs comes from the difference in projection pattern onto CA1 dendrites. The dendrites with CA3 synapses are generally not as ramified as are those with EC synapses. Therefore, similar activation patterns in CA3 would have less distinct effects on action potential initiation. Another factor could be the difference in the connectivity between CA3 and CA1 compared to EC and CA1 in the model. Each CA3 cell targeted 3697 CA1 principal cells whereas each EC cell targeted only 1000 CA1 principal cells. Because of the greater divergence factor, differences in CA3 activity patterns would be expected to be more averaged out in CA3 spike activation patterns compared to differences in EC activity patterns. Testing of those conjectures is computationally tractable and could be performed in future studies.

The predictions of our study regarding transformations of CA3 and EC inputs in CA1 could be tested by simultaneous recording of CA1 neurons and their presynaptic CA3 and EC neurons in behaving rats (Fyhn et al., 2007; Colgin et al., 2009). Colgin and colleagues (Colgin et al., 2009) showed that some CA1 principal cells predominantly process information from EC cells with inputs synchronized with fast gamma (65–140 Hz). Other CA1 principal cells predominantly process information from CA3 cells and do so in the slow gamma range (25–50 Hz). Our results predict that provided the same (relative) difference between CA3 spike patterns and EC spike patterns the Hamming distance between the corresponding CA1 output spike patterns will be greater for the EC patterns. Future simulation studies should allow us to predict the changes in the input-to-output transformation in CA1 due to altered inhibition in CA1 as might occur in schizophrenia (Behrens and Sejnowski, 2009). Similarly, predictions could be generated regarding reduction of spine density in animal models of Alzheimer's disease (Perez-Cruz et al., 2011). The changes in the transformation of inputs-to-outputs in CA1 will predict alterations in learning and retrieval of information in these disorders.

The results of our study relate mostly to the initial CA1 responses to inputs. These responses occur before synaptic plasticity and information exchange between CA1 inhibitory and principal cells build up. Initial hippocampal responses to stimuli can have significant impact on behavior. For example, they may initiate different responses to startling events (Lin et al., 2006). Our study raises questions regarding what information prevails in determining those responses.

### Conflict of interest statement

The authors declare that the research was conducted in the absence of any commercial or financial relationships that could be construed as a potential conflict of interest.
